# Nonequilibrium Population Dynamics of Phenotype Conversion of Cancer Cells

**DOI:** 10.1371/journal.pone.0110714

**Published:** 2014-12-01

**Authors:** Joseph Xu Zhou, Angela Oliveira Pisco, Hong Qian, Sui Huang

**Affiliations:** 1 Institute for Systems Biology, Seattle, Washington, United States of America; 2 Kavli Institute for Theoretical Physics, University of California Santa Barbara, Santa Barbara, California, United States of America; 3 Systems Biology Program, Faculty of Life Sciences, University of Manchester, Manchester, United Kingdom; 4 Department of Applied Mathematics, University of Washington, Seattle, Washington, United States of America; 5 Institute for Biocomplexity and Informatics, University of Calgary, Calgary, Alberta, Canada; Hungarian Academy of Sciences, Hungary

## Abstract

Tumorigenesis is a dynamic biological process that involves distinct cancer cell subpopulations proliferating at different rates and interconverting between them. In this paper we proposed a mathematical framework of population dynamics that considers both distinctive growth rates and intercellular transitions between cancer cell populations. Our mathematical framework showed that both growth and transition influence the ratio of cancer cell subpopulations but the latter is more significant. We derived the condition that different cancer cell types can maintain distinctive subpopulations and we also explain why there always exists a stable fixed ratio after cell sorting based on putative surface markers. The cell fraction ratio can be shifted by changing either the growth rates of the subpopulations (Darwinism selection) or by environment-instructed transitions (Lamarckism induction). This insight can help us to understand the dynamics of the heterogeneity of cancer cells and lead us to new strategies to overcome cancer drug resistance.

## Introduction

**Figure 1:**
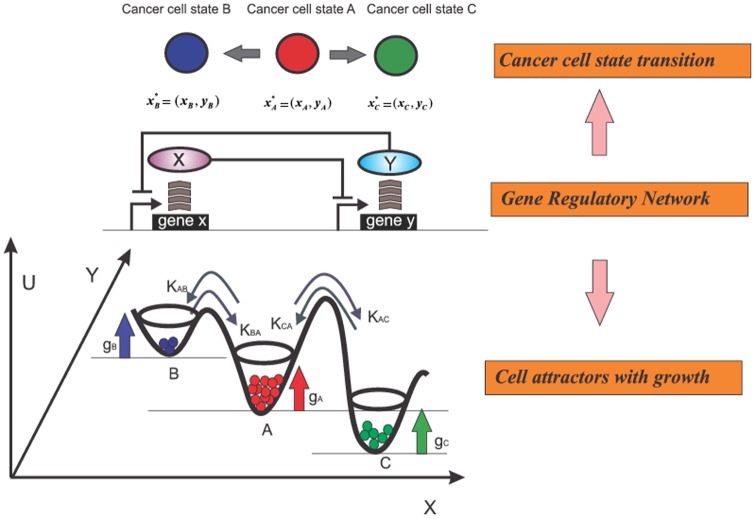
Schematic illustration of a cell population dynamics with three distinct cell states. **A.** Three cell states 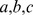 with distinct gene expression 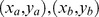 and 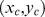. **B**. The gene regulatory circuit of X and Y determines three cell states 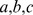. **C**. Each state is associated with a growth rate 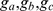 respectively. Three states transition to each other with the interconversion rates 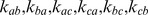.

During cancer progression, alike development and homeostatic activities, tumor cells undergo phenotypic changes such as cell differentiation, immune activation during inflammatory response, or epithelial to mesenchymal transition (EMT). A switch of cell state is driven by genome-wide gene expression changes that follow characteristic patterns. For instance, in response to a signal that promotes differentiation, a population of immature progenitor cells expresses proteins *X and Y*, which are associated with the differentiated state (“differentiation marker”) and needed for the physiological functions of the differentiated cells (Fig. 1A). The gene regulatory network (GRN) coordinates the changes in the expression levels of the genes that implement specific phenotypic states of cells. GRN describes how the regulatory genes control one another's expression in a predetermined manner, which is encoded in the genome (Fig. 1B). We can thus represent a cell state 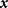 by its expression pattern 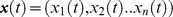 of the *n* genes where 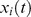
is the expression activity of gene locus 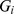 quantified at the level of the genomic locus, either in the form of transcripts or proteins. Due to inherent nonlinearities of the dynamics of such networks, a rich structure of the state space (space of all configurations of 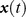) with multiple attracting regions (“multistability”  =  coexistence of multiple stable states) arises such that each attracting domain maps into a distinct cell phenotype or behavior, as shown in Fig. 1C. The basins of attraction compartmentalize the network's state space and give rise to disjoint stable states 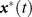 – capturing essential properties of cell types [1]. The theory, first proposed more than 50 years ago [2], [3], that (high-dimensional) “attractors” represent the various cell types of the metazoan organisms built the foundation to understand cell state transition and cell population dynamics.

A cell is the elementary unit in a population whose birth, death and transformation events underlie the population dynamics. Many studies describe the cellular transition using a master equation either in the discrete formalism, like Boolean networks [4], [5], or in the continuous formalism of ordinary differentiation equations (ODEs) [6]–[8]. The assumption of mass conservation is generally used in models inspired by rate equations in chemistry. However, it needs to be taken into account that cellular multiplication violates the mass conservation. The departure from mass conservation spontaneously change the probability density 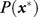 in absence of influx/efflux to/from state 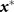. This notion is of central importance to understand tissue formation since the cell population dynamics become non-equilibrium dynamics. The ratio between fractions of cells corresponding to different phenotypes no longer unconditionally approaches a steady state, considering both cell proliferation and cell transition. Together with the transition rate, the net cell growth (proliferation minus death) also changes the abundance of cells in attractor state 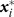 and consequently affects the occupied ratio of attractor states, changing the overall tissue conformation.

In population biology, notably in the study of evolution dynamics, many researchers have modeled heterogeneous populations of distinct species that differ in “fitness” [9]. One closely related mathematical theory of cell population dynamics is Luria-Delbrück theory, initiated by Luria and Delbrück and extensively developed later by Lea and Coulson, Kendall, Bartlett, Armitage and Doll and many others [10], [11]. Typically in these models, population heterogeneity is due to the diversity of genotypes produced by genetic mutations instead of multistability and non-genetic (“epigenetic”) transitions between multiple attractor states. These classical evolution models of cell populations have played an important role in the analysis of the somatic evolution of cancer cells, thought to be the major driver of cancer progression [9], [12]. However, these models tacitly assume a one-to-one mapping between genotype and phenotype and assume random genetic mutations as the mechanism for cell phenotype switching.

Recent advances in mammalian cell reprogramming and cell transdifferentiation have underscored the importance of multistability and non-genetic cell state transitions resulting in non-genetic cell population dynamics [13], [14]. Considering such non-genetic dynamics will lead to models that differ from classical population genetics models in the following points:

- ***Reversible*** **state transitions**: these transitions are often approximately symmetrical while mutations in the traditional model are typically irreversible;
- ***Frequent*** **state transitions**: transition rate often is in the same time scale as division time or even faster, while mutation rate per locus is much slower than the division rate.
- **Transitions are not strictly non-Lamarckian**. They can be induced in a controlled (purposeful) or uncontrolled manner while mutations are randomly directed and their rates not easily tunable.

The key focus of this paper is the dynamics of the cellular composition of a growing cancer cell population. More specifically, we study the dynamics of the relative abundance of distinct cancer cell subtypes. We also discuss the conditions for a cancer cell population to maintain the fixed ratio and distinct cell types. Finally we study how transition and growth rates influence the subpopulation ratio when cell population reaches equilibrium. Since the true novelty of this research is to introduce the state-dependent growth rate to cell state transition, in general this model can be used to describe the cell differentiation during embryogenesis, or any cell population dynamics in which growth and transition both play important roles in the same scale.

## Cell Population Model for Transition and Growth Dynamics: Two-Phenotypes

We start with a simple model of cancer cell population with two-phenotypes. We assume that bimodal expression levels 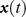 of markers 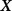 can modulate the growth rate. The discretization of a cell population with continuously distributed gene expression levels 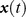 into two states, 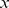 and 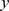, is appropriated to capture the characteristic population dynamics. Nonlinear dynamical systems usually have stable steady states (attractors) and the system should return to attractors under reasonably small perturbations. The dynamics of returning to the attractors or re-establishing the equilibrium after perturbation is usually a key property of nonlinear dynamical systems. It helps us to understand the mechanism of many interesting biological phenomena in biology, such as polymorphism, homeostasis etc. Here we first establish the conditions for the co-existence of the two phenotypes by characterizing both the existence of a steady-state ratio of these two types and the dynamics underpinning the re-establishment of the equilibrium.

### Elementary model: two-phenotype cell population dynamics

We consider the dynamics of a cell population with two interconverting states (“subpopulations”) 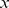 and 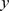 with relative abundances 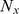 and 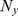, which have their own birth rates 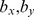, death rates 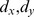 and state transition rates 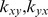. The net growth rates (the quantities readily measured) are 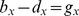 and 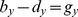, respectively. The cell population dynamics including both cell growth and state transition are described by the corresponding set of ODEs: 
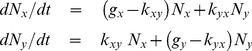
(1)


Eq. (1) matrix representation is given by:
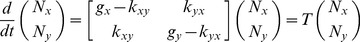
(2)where 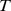 is a matrix with both growth and transition terms. It is important to emphasize that only the net growth rate can be reliably measured in cell culture. If 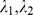 are eigenvalues of 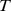 in Eq. (2), the general solution of this linear ODE system is:
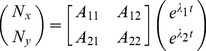
(3)


The long-term dynamic behavior of this dynamic system is then determined by the two eigenvalues 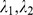. There are two distinct behaviors if the eigenvalues either have opposite signs or the same sign. When the two eigenvalues have opposite signs, i.e. 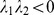, the growth term associated with the negative eigenvalue decays exponentially. In this situation the two cell populations have essentially the same growth rate, defined by the positive eigenvalue with only different pre-factors *A_11_ (when* 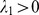) and *A_22_* (*when* 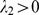). This indicates that only one subpopulation can survive independently while the second subpopulation lives as the derivative of the other one.

When the two eigenvalues have the same signs, i.e. 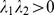, we need to consider whether they are either positive or negative. If both eigenvalues are positive, the two subpopulations survive together. In the long term both populations will grow with the same rate, defined by the larger eigenvalue. Finally, if both eigenvalues are negative, then both subpopulations become extinct together. Mathematically, since the product of the two eigenvalues is the determinant of the matrix *T*, the condition for the two eigenvalues having the same sign can be written as:
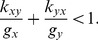
(4)


Therefore, if the growth rates for both phenotypes are much greater than the state conversions, i.e. 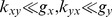, then the two subpopulations can both survive on their own. However, if one of the two populations has a transition rate larger than its division rate, this population can only survive as a “***derivative***” of the other (as it depends on the “backflow” from the other). This simple mathematical observation has consequences in non-genetic drug resistance (persistors) [15], [16]. If the conversion rates of both subpopulations are much larger than their respective growth rates, the distinction of the two discrete cell phenotypes becomes blurred. Interestingly, in this case none of the subpopulations can survive alone. We can consider these two populations as a single one with a mean growth rate of 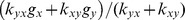. Thus, by examining transition rates in regimes never considered in mutation-based population dynamics (because mutations are rare), we enter a dynamical regime that is relevant for cell population dynamics in which non-genetic phenotype conversions dominate.

We can determine the population ratio 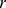
between the two subpopulations in this regime. Since in the long term both populations growth is given by the term with the larger eigenvalue, i.e. 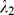, their ratio is essentially *A_21_/A_22_*. The dynamics of 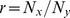 follow 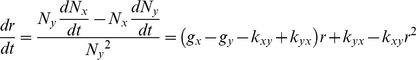
(5)


Therefore, the steady-state ratio fraction of 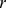 is
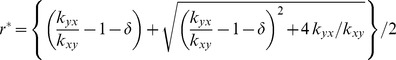
(6)in which we denote 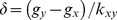 as the differential net growth rate of 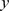 with respect to that of 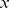 divided by the transition rate from 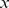 to 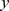. While the population ratio 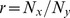 becomes stationary, both populations of 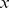 and 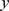 can grow indefinitely. This result deviates from classical population dynamics in which the coexistence of two populations of different growth rates is not stable due to one-direction conversion (mutation). In fact, recent work on clonal (isogenic) cancer cell populations showed that they typically consist of several interconverting discrete sub-populations associated with biologically relevant functional properties, such as stem-like behavior, drug–efflux capability [14], [17] and differentiation [13]. The exponential growth at a constant ratio r* also agrees with the observation that cells which are continuously passaged in cell culture keep the fixed ratio between sub-types; the total population 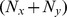 growth rate is then given by:
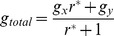
(7)


The question now is: Can we quantify the different influences on the observed cell fixed ratio from the growth and transition rates? A possible biological interpretation is that changes in 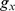 and 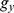 relative to each other represent differential fitness in a given environment, which could promote Darwinian selection. Along the same line, changes in 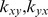 can represent Lamarckian instruction in the sense that a given environment may impose differential transition rates between different phenotypes. This offers a simple mathematical framework to describe the relative contribution of Darwinian selection and Lamarckian instruction in shifting population ratios during tumor progression under chemotherapy.

### Re-equilibrium of two-phenotypes cell population

It is easy to obtain the time-course for the dynamics of the re-equilibrium of the subpopulations by finding the integral solution for Eq. (5). For the convenience of integration, we change the variable 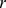 to 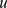 and integrate it from the initial cell population ratio 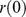 to the ratio 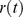 at any arbitrary time 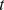:
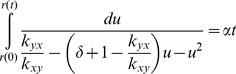
(8)


Since 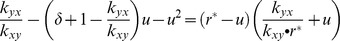, where 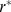 is given by Eq. (6), we have



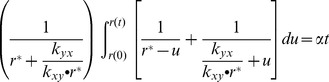


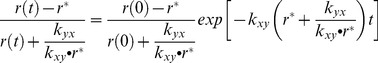
(9)

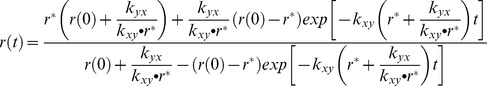



This result suggests that the re-equilibrium time is in the order of 
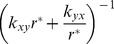
. We can also benchmark this equation with two extreme cases whose transition rates are obvious. The initial rate for re-equilibrium starting with a pure subpopulation 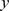, i.e., 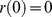, is given by:
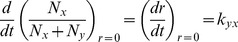
(10)while the initial rate for re-equilibrium starting with pure 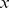, i.e., 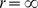, can be written as:
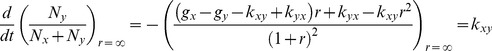
(11)


In the general case, re-equilibrium rate is a dynamic process that combines both Eq. (10) and (11). Eq. (5) also predicts that there are two types of re-equilibrium dynamics. The right-hand-side of Eq. (5) reaches its maximum at
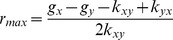
(12)with the rate
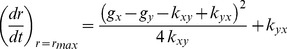
(13)


**Figure 2:**
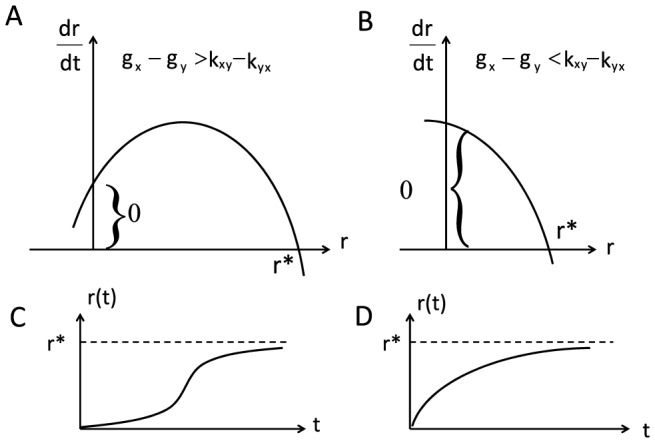
The re-equilibrium of the cell subpopulations after cell sorting can have different dynamical behaviors. **A.** Cell differential growth rates are bigger than cell differential transition rates. The time derivative of cell ratio is non-monotonic before reaching the fixed ratio 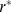. **B**. Cell differential growth rates are smaller than the cell differential transition rate. The time derivative of cell ratio is monotonically decreasing before reaching the fixed ratio 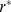. **C**. Cell re-equilibrium dynamics is sigmoidal for the condition shown in A. **D**. Cell re-equilibrium dynamics is logistical for the condition shown in B.

Therefore, if 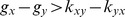, i.e. the difference of growth rates is larger than the difference of transition rates, one expects that the re-equilibration can be described by a sigmoidal curve along time. The rate for re-equilibrium increases with time until 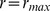, followed by a decreasing rate. This is shown in Fig. 2A and 2C. However, if 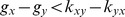, i.e. the difference of growth rates is smaller than the difference of transition rates, the rate of re-equilibrium dynamics, starting at *r* = 0, decreases monotonically with time. In this case, the time course of re-equilibration follows a exponential saturation kinetics (monotonically increasing before reaching the saturation), as shown in Fig. 2B and 2D.

## Cell Population Model for Transition and Growth Dynamics: *M*-Phenotypes

During the development of multicellular organisms, usually more than two cell types are formed. This introduces qualitatively new properties not seen in the classical two-state transition model. To study this phenomenon, we extended our mathematical formalism above to 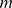 cell phenotypes:
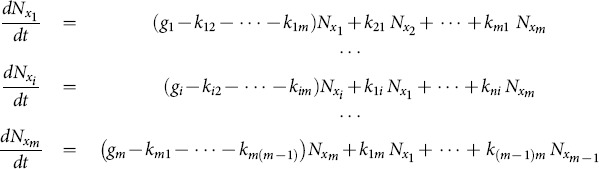
(14)


Here 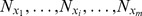 are the respective number of cells of 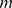 phenotypes in the cell population. Each phenotype has a respective growth rate 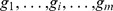; 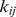 are the state transition rates from cell phenotype *i* to phenotype *j*. Since this is a linear system, the right-hand-side can be decomposed as a sum of a diagonal matrix and a Markov matrix. The matrix form of Eq. (14) can be written as: 
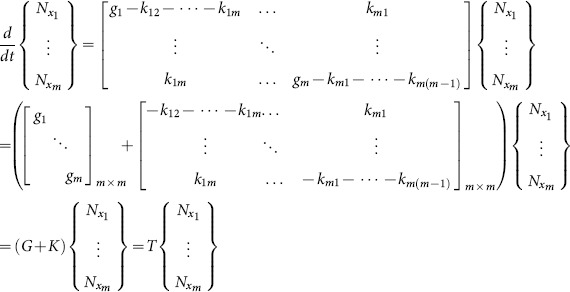
(15)


Here 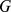 is a positive diagonal matrix with growth rates 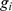 and 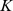 is a Markov matrix consisting of transition rates 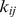. The sum of each column of 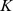 is zero due to the flux conservation principle. This means that there are at least one zero eigenvalue for the Markov matrix, which guarantees that there exists a steady state 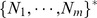 if the system is a transition-only dynamical system (*G* = 0). If growth rates are not zeroes, there will be a steady state for cell relative ratio instead of an absolute cell number 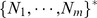, shown in the following equation. We can then establish the mathematical relationship between the growth and transition rate, which is necessary to maintain distinct epigenetic phenotypes and the fixed cell ratio. If the matrix 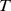 has 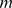 eigenvalues 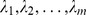, then the general solution of Eq. (15) is 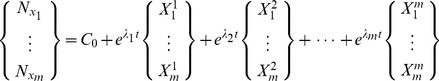
(16)


Here 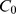 are constants determined by the initial conditions of the dynamical system. Each subpopulation 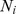 is the linear combination of some exponential growth functions. Therefore, there is no nontrivial steady-state 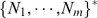 as it does in the case of the transition-only dynamics, i.e. this linear system represents an ideal world with ample nutrients in which cells can grow indefinitely.

The co-existence or co-extinction of ***m*** distinct cell subpopulations requires 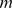 eigenvalues that satisfy either 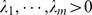 or 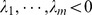. Since we want the cell population to maintain the distinct phenotypes, the growth rates for all phenotypes need to be much larger than the sum of the state conversions: for 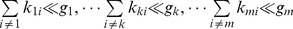, the 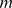 subpopulations can survive on their own. However, if one of the 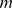 populations has a transition rate larger than its growth rate, this population can only survive as a “*derivative*” of the others (as again it depends on the “backflow” from the others). If, on the other hand, all subpopulations have conversion rates much larger than their growth rates, the distinction of multiple discrete cell phenotypes becomes blurred. In this situation there will be only one cell population with subpopulations quickly transitioning between each other, and because of that none of the subpopulations can survive on its own.

## Biological Examples and Interpretation

**Figure 3:**
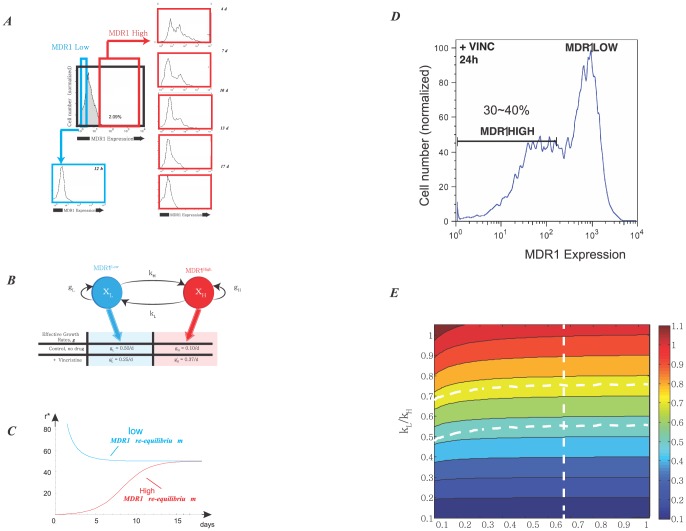
HL60 cell population dynamics. Leukemia cell line HL60 has two subpopulations, MDR^High^ and MDR^Low^, based on their abilities to retain CalceinAM fluorescent dye (flow cytometry profiles), as measured by flow cytometry. The flow cytometry histograms correspond to a snapshot of the cell population at a given time point. In this particular case the parameter is the accumulation of a fluorescent dye, CalceinAM, which works as a surrogate for ABC transporters activity and multidrug resistance: if the cells retain the dye, ABC transporters are not active and the cell is sensitive to drug; if the cells do not accumulate the dye, ABC transporters are active and the cell is resistant to drug treatment. **A**. In the absence of drug the two subpopulations co-exist at a stable cell ratio, MDR^High^ = 2% and MDR^Low^ = 98%. **B**. When the cells are treated with 10 nM of vincristine for 72 h the proportions change to MDR^High^ = 40% and MDR^Low^ = 60%. For further details please refer to Pisco et al [17].

In this section we are going to exemplify the concepts described above using experimental data of cancer cell population dynamics. It is well known that individual tumors harbor numerous cellular phenotypes and each phenotype has different biological properties, such as growth rates, migration abilities and drug responses. For example, cancer stem cells are usually associated with tumor-initiation, metastasis and drug resistance. One of the biggest challenges that cancer research faces is drug resistance. There are two hypotheses about the origin of the resistance to chemotherapy or radiotherapy [18]. The first hypothesis proposes the pre-existence of drug-resistant subpopulations, which are able to survive in the presence of the drug and to expand during and after the drug treatment. The second hypothesis assumes that cancer cells are phenotypically plastic and capable of transiting between drug-sensitive and drug-resistant states. In our previous studies [19] we have modeled the response of acute leukemic cells HL60 to chemotherapeutical drugs to distinguish these two possible mechanisms of drug resistance. Given that constitutive expression of *ABC* transporters is usually associated with multidrug resistance, we measured expression and activity of the *ABCB1/MDR1* transporter before and after drug treatment. We found that within the HL60 cell population there are two subpopulations, MDR1^Low^ and MDR1^High^, which respectively correlate with low or high survival in the presence of drug (Fig.3). The two subpopulations can spontaneously interconvert among themselves, with a stable co-existence ratio of 98∶2 (MDR1^Low^: MDR1^High^) without drug (Fig.3A) and 60∶40 with drug (Fig.3B). Our results have showed that the response to drug was predominantly controlled by the change in transition rate, rather than by differential growth rates. Here we expanded our conceptual approach to study state transitions in a breast cancer experimental system with three possible phenotypes [20].

### Three-phenotype transition between breast cancer stem cells and differentiated cancer cells

Breast cancer cell lines SUM159 and SUM149 show three different behaviors, based on putative cell-surface markers: stem-like cells (CD44^high^ CD24^neg^ EpCAM^low^), basal cells (CD44^high^ CD24^negative^ EpCAM^negative^) and luminal cells (CD4^low^ CD24^high^ EpCAM^high^) [20]. Gupta et al [18] have shown that SUM159's cell population is predominantly basal, with an associated fixed cell ratio of 97.3% basal (B), 1.9% stem (S) and 0.62% luminal cells (L). On its turn, cell line SUM149 predominately consists of luminal cells, with a respective proportion of 3.3% basal (B), 3.9% stem (S) and 92.8% luminal cells (L). In the study, the authors demonstrated that if the three different cell states are FACSorted based on their surface markers, and the relatively pure cell subpopulations were allowed to grow in regular culture conditions, all sorted pure cell subpopulations quickly recovered the initial cell population ratio. It is then important to ask why tumors maintain this heterogeneity with fixed ratio and what are the mechanisms leading the quick relaxation of the sorted cell sub-populations back to the initial ratio.

Gupta et al [20] used a Markov model to describe the re-equilibrium dynamics of cell subpopulations. Although the Markov model is able to explain the existence of stable cell fractions' ratio and can also capture the dynamics of the cell states, there are two disadvantages when comparing to a ODE model. First, the Markov model re-scales the total cell population to 1 at each time step, masking the influence of different growth rates of subpopulations. The probability of remaining in the same state in the Markov model, which corresponds to the growth rate in ODE model, will give the cell ratio for steady state conditions but cannot predict the effective growth rate of the whole cell population. Second, re-equilibrium is guaranteed in a Markov process as long as the probability of transitions is conserved (e.g., each row of the probability transition matrix add up to 1). However, there are some subtle connections between the growth rates and the transition rates, which are missed by the Markov model, such as that the growth rate and the transition rate need to satisfy condition in Eq. 6 to reach the steady state, and the conditions for co-existence or derived existence of different subpopulations. The quantitative model developed in Section 3 is used to address these questions. We are able not only to examine the requirement for reaching a fixed ratio between different cell subpopulations, but also to characterize the condition of its existence in a multiple-cell-type and continuously growing cell population. If we assume for the subpopulations 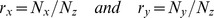, by the chain rule of differentiation 
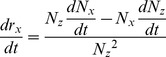
, 
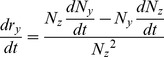
, the dynamics of 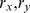 follow: 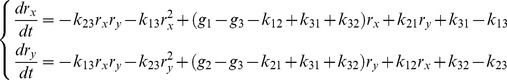
(17)


In order to get the steady state of cell ratios, we set 
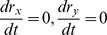
 in Eq. (17) above. This leads to a dual quadratic equation, which has no analytical solution in general. However, this can be solved with numerical methods.

Even if the population ratios 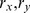 become stationary, the absolute cell number of the subpopulations, 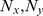 and 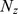 can increase indefinitely. In the situation where growth rates are smaller than transition rates 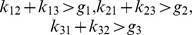, the transition matrix is predominantly a Markov matrix that satisfies the flux conservation principle as it has only one positive eigenvalue while all others are negative. All cell subpopulations grow exactly at a single growth rate 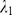 and their ratios will be determined by the initial constants 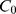 (see Eq. (16)). Therefore, the subpopulations virtually correspond to the same cell type with different expression levels for biomarker *X* and none of them can survive as an independent cell type. In practice, such rapid transitions will manifest as fluctuations of gene expression profiles, contributing to the observed population heterogeneity in snapshots. As explored in Pisco et al. [19], changes in 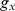 and 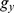 can represent differential fitness in (mutation-less) Darwinian selection whereas changes in 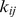 can represent Lamarckian instruction.

By putting together the growth-transition linear ODE model of Eq. (14) with the steady-state population fraction of 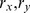 of Eq. (17), we can estimate the growth rates 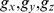 and the state transition rates 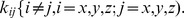 There are in total nine unknown variables (3 growth rates and 6 transition rates) but we have only 2 equations (with 
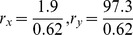
 for SUM159 cell lineage and 
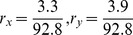
 for SUM149 cell lineage). The solutions are undetermined because there were no measurements for growth rates or re-equilibration time for each cell population. However, since the cell fraction ratios are experimentally measured at day 0 and at day 6 [18], we can run parameter scanning to find the values which fit the ratios best at day 0 and day 6. The basic procedure of parameter scanning include two steps: first, the range of scanning values and the increments are estimated for each parameter; second, a large parallel trial-and-error test was performed to find the parameters that better fit the re-equilibration data.

**Figure 4:**
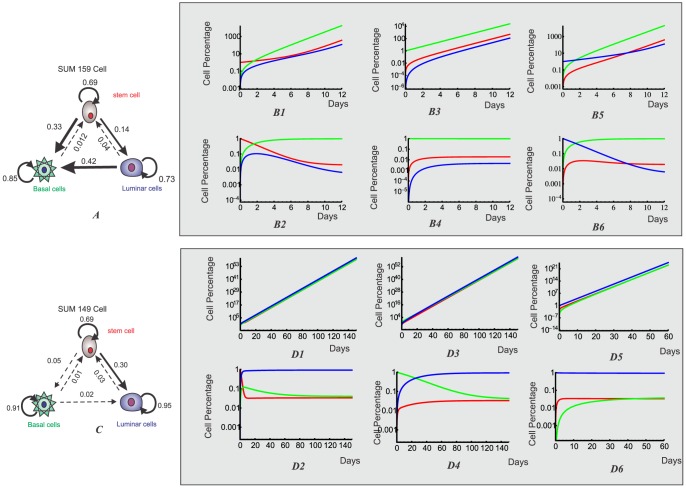
Three-phenotypic breast cancer cell population dynamics with both growth and transition from model simulation. **A.** Illustration of cell growth and transition for breast cancer cell line with three distinct cell phenotypes: luminal cell, basal cell and mammary stem cell. **B1-B6**. After FACS sorting, each isolated subpopulation of cell line SUM159, stem-like, basal and luminal cells, re-equilibrate to the stable cell-state ratio 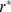. Upper panels are the dynamics for cell numbers of three subpopulations; Lower panels are the dynamics for the cell ratios of three subpopulations. **C1-C6**. After FACS sorting, each isolated subpopulation of cell line SUM149, stem-like, basal and luminal cells, re-equilibrate to the stable cell-state ratio 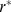. Upper panels are the dynamics for cell numbers of three subpopulations; Lower panels are the dynamics for the cell ratios of three subpopulations.

The cell population model with growth and inter-conversion is shown in Fig. 4A. The growth rates and transition rates for SUM159 cell line, obtained from the parameter scanning, are listed in Table 1. The eigenvalues 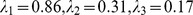 are the values that provide the best fit of the experimental data. As we showed in Section 3, the distinctive cell subpopulations and the co-existence or co-extinction of them requires three eigenvalues to satisfy all 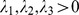. Therefore, these three cell types can co-exist. Another criteria is to check whether the growth rates for all phenotypes are much faster than the sum of the state conversions, i.e.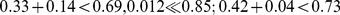, such that the three subpopulations can all survive on their own. The same procedure was applied for the SUM 149 cell lineage to obtain the growth rates and the transition rates, as shown in Table 1. By computing the eigenvalues 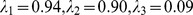 we concluded that the subpopulations of SUM149 can also co-exist. When we check the relationship between the growth rates and the transition rates, we have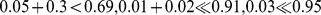, showing that the three subpopulations can all survive on their own as well.

**Table 1:** The estimated values of growth rates and transition rates for breast cancer cell lines SUM159 and SUM149 to reach stable cell fraction ratio 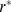 after FACS sorting.

	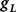	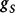	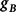	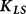	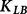	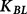	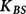	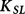	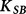
**SUM159**	0.73	0.69	0.85	0.04	0.42	0	0.012	0.14	0.33
**SUM149**	0.95	0.69	0.91	0.03	0	0.02	0.01	0.30	0.05

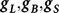
 are growth rates of the subpopulation Luminal, Basal and cancer stem cells. 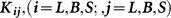 are transition rates between two breast cancer cell subpopulations.

The dynamics for re-equilibrium of SUM159 cell line are shown in Fig. 4B1-B6. We can clearly see that after cell sorting each cell sub-population quickly re-equilibrated to the stable steady-state ratio r* =  *N_X_*∶*N_Y_∶N_Z_*≈1.9∶97.3∶0.62 within 12 days. However, since SUM149 cell line generally has much slower transition rate, the population slowly re-equilibrated to the stable steady-state ratio until the end of the 140 days time-course, as shown in Fig. 4C1-C6. Our computational results for both cell lines qualitatively agree with the experimental data and the Markov model simulation results presented in Gupta *et al* [18], which showed that SUM159 cells quickly returned to the equilibrium while SUM149 cells were far from reaching the equilibrium at day 6. Moreover, the ODE model revealed more information about cancer population dynamics. First, the ODE model computes the results for the cell population in real number, which is the gold standard for cancer drug evaluation. Second, we decided whether each cancer subpopulation can survive independently or as the derived one from other subpopulations. This finding has important implications during drug design, as it can inform which population is the more appropriated to target.

## Discussion and Conclusion

### Influence of the growth term in the cell population dynamics

We discuss the consequences of introducing the growth rate term for the steady state transition ratio 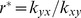 in our two-phenotype model. Here 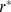 is the ratio of cell numbers in the phenotype space, where each phenotype is determined by a state 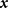 of the cell's molecular network. The dynamics of state 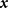 are captured by the transition rates 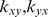 as well as the growth rates 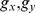, which vary from state to state. It offers both a rationale and a framework to understand the genotype-to-phenotype mapping problem in complex organisms [1], [21]–[26]. If the growth rate is not included in Eq. (1) the steady state is simply given by the ratio 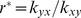. The re-equilibrium is guaranteed in any situation, independent from the initial population ratio and from the transition rates. When the growth rate is considered, there is no unconditional re-equilibrium for any transition rate. The growth rates 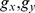 and transition rates 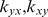 have to satisfy the condition defined in Eq. (6). Also, to maintain two distinct cell subpopulations, it needs to satisfy the condition given by Eq. (4), i.e. transition rates 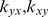 have to be much smaller than the growth rates 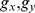; otherwise, we will be in the presence of a cell population with two indistinguishable subpopulations that can quickly transit between each other. In our ODE model for the breast cancer cell population dynamics, transition rates are indeed much smaller than the growth rate to guarantee independent existence of each cell population.

### Selection vs. instruction dualism

By uniting the dynamics of reversible transitions between distinct cell phenotypes with their growth rates, we can capture the contributions for the evolution of the cell population from both Darwinian selection and Lamarckian induction. This has fundamental implications because evolutionary dynamics is commonly used to explain the changes that happen at tissue level during tumor progression [27]–[33]. Since an attractor state confers a discrete, stable phenotype that can be inherited across cell divisions to future generations, we enter the new realm of non-genetic (mutation-less) Darwinian evolution. The fact that a cell phenotype (attractor) transition can be triggered by environmental perturbation also permits the consideration of Lamarckian evolution in cell populations during tumor progression, for which there are increasing evidences [19], [20], [34]–[40]. The general formalism combining state transitions and differential growth also reconciles the old debate between the selective (“stochastic” or “permissive”) vs. the instructive modes of cell fate determination during development [41]–[44]. Moreover, directed cell-to-cell interactions between cells in distinct states 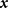 mediated by the expression of communication signals (cytokines), which are a function of 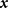, and affect proliferation and phenotype switching, can also be readily considered to incorporate non-cell autonomous effects in population dynamics [45].

The study of tissue change at the granularity of cell population dynamics also has direct impact on our understanding of the elementary principles of evolution. The macroscopic change of a population's property X in a particular environment *S* in one direction can in principle be achieved by two mechanisms. Mechanism (i) is selection - in the presence of S, cells that “happen” to be state 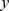 have a higher growth rate (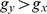). There is no actual change of gene expression state in any cell as this is a population level change. A core element of this process is randomness because the direction of change comes from the environment S. Mechanism (ii) is instruction: S induces a gene expression pattern change 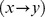 in individual cells that confers a new phenotype. This is an individual cell level event, a change of the molecular network state of a cell. In evolution this dualism obviously maps into that of Darwinism vs. Lamarckism (in the case when the S-induced property endures and confers advantages in coping with S). Here we deal with a scheme of change and not its material cause [32]. Eq. (6) shows that fundamentally, in terms of schemes, Lamarckism and Darwinism are just the two sides of the very same dynamical process: (1) for 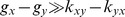, (i.e. the difference of growth rates is far bigger than the difference of transition rates) Darwinian mechanism dominates in the dynamics of cell population ratio; (2) for the opposite case, when 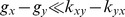, Lamarckian mechanism determines the cell population ratio. Depending on the “half live” of the new induced state (relative stability) we would have inheritance of an acquired trait for at least several generations. In general the Darwinian scheme is invoked by default to explain cell differentiation, developmental processes and cancer origination. We propose that the alternative Lamarckian scheme also needs to be taken into account in the sense that either scheme has to be verified or falsified experimentally.

### Conclusion

In this paper we provide a mathematical framework of population dynamics that considers both distinctive growth rates and intercellular transitions between cancer cell populations. Our mathematical framework showed that both growth and transition influence the ratio of cancer cell subpopulations, and transition's role is even stronger. We derived the condition that different cancer cell types can maintain distinctive subpopulations and we also explain why there always exists a stable fixed ratio after the cell sorting based on certain surface markers. While Lamarckism has little role (if any at all) to play in organism evolution, our model is the first attempt to demonstrate that in principle Lamarckism and Darwinism (as an evolved adaptive response of a cell population) are two different sides of the same coin, two extremes of the same spectrum of behavioral schemes. This not only applies to the study of cancer cell dynamics, but also helps our understanding of the emergence of drug resistance in anti-cancer therapy.
