# Correction: Nonequilibrium Population Dynamics of Phenotype Conversion of Cancer Cells

**DOI:** 10.1371/journal.pone.0118133

**Published:** 2015-03-26

**Authors:** The PLOS ONE Staff

The image for Fig. 1, “Schematic illustration of a cell population dynamics with three distinct cell states,” is incorrect. Please see the corrected here Fig. 1.

**Fig 1:**
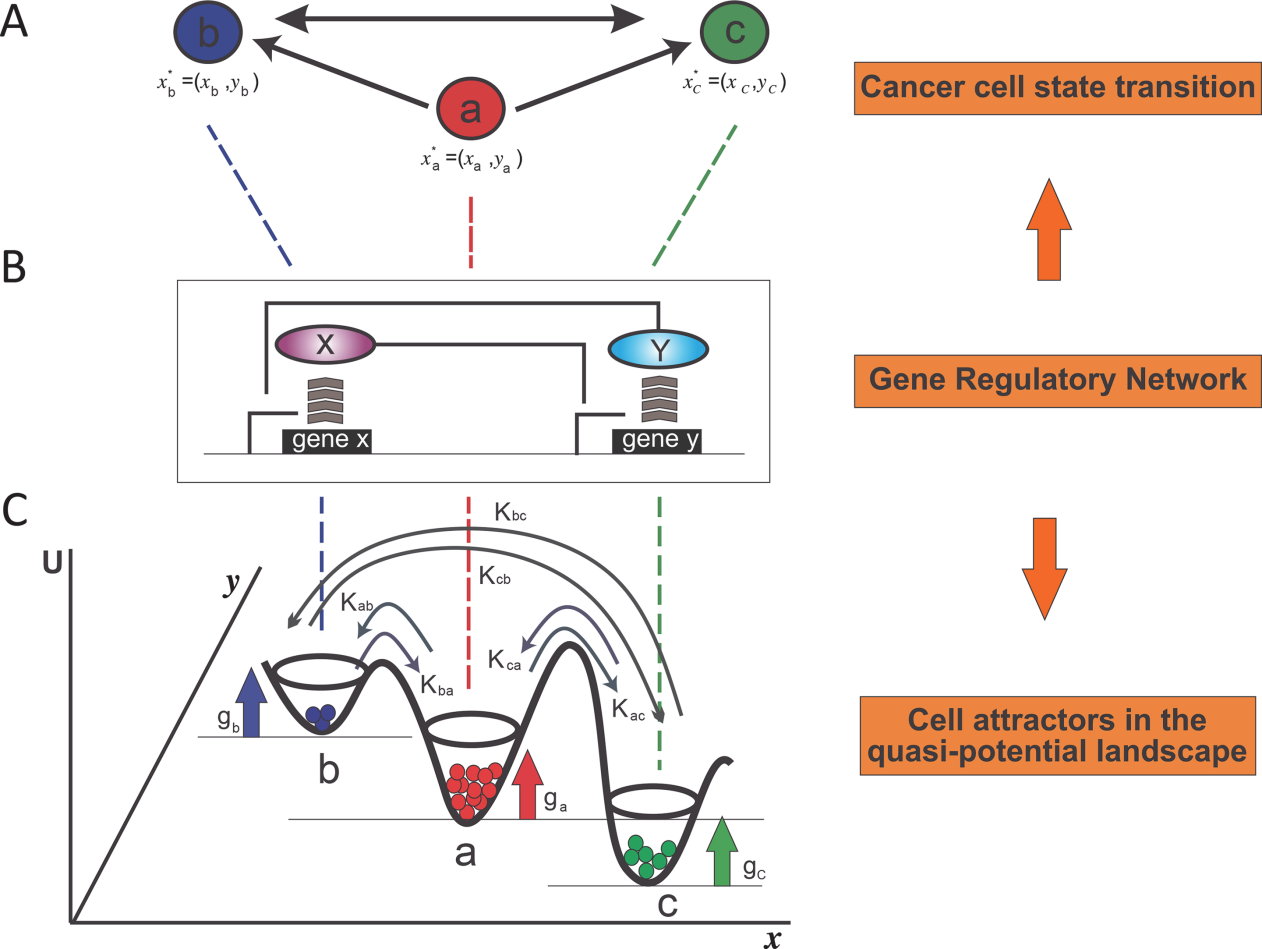
Schematic illustration of a cell population dynamics with three distinct cell states. **A.** Three cell states *a*,*b*,*c* with distinct gene expression (*x*_*a*_,*y*_*a*_),(*x*_*b*_,*y*_*b*_) and (*x*_*c*_,*y*_*c*_). **B**. The gene regulatory circuit of X and Y determines three cell states *a*,*b*,*c*. **C**. Each state is associated with a growth rate *g*_*a*_,*g*_*b*_,*g*_*c*_ respectively. Three states transition to each other with the interconversion rates *k*_*ab*_,*k*_*ba*_,*k*_*ac*_,*k*_*ca*_,*k*_*bc*_,*k*_*cb*_.

There is an error in the first sentence of the seventh paragraph of subsection “Elementary model: two-phenotype cell population dynamics” of section “Cell Population Model for Transition and Growth Dynamics: Two-Phenotypes”. The correct sentence is: We can determine the population ratio *r* between the two subpopulations in this regime.

The image for Fig. 3, “HL60 cell population dynamics,” is incorrect. Please see the corrected here Fig. 3.

**Fig 3:**
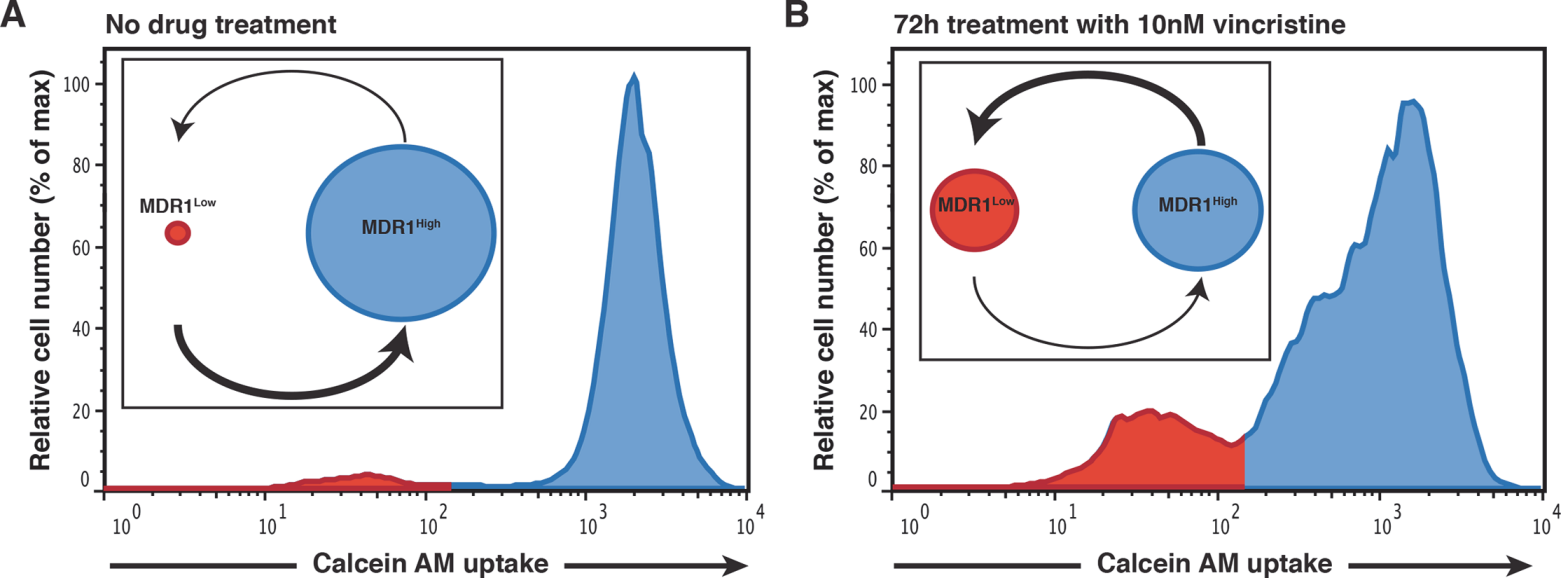
HL60 cell population dynamics. Leukemia cell line HL60 has two subpopulations, MDR^High^ and MDR^Low^, based on their abilities to retain CalceinAM fluorescent dye (flow cytometry profiles), as measured by flow cytometry. The flow cytometry histograms correspond to a snapshot of the cell population at a given time point. In this particular case the parameter is the accumulation of a fluorescent dye, CalceinAM, which works as a surrogate for ABC transporters activity and multidrug resistance: if the cells retain the dye, ABC transporters are not active and the cell is sensitive to drug; if the cells do not accumulate the dye, ABC transporters are active and the cell is resistant to drug treatment. **A**. In the absence of drug the two subpopulations co-exist at a stable cell ratio, MDR^High^ = 2% and MDR^Low^ = 98%. **B**. When the cells are treated with 10 nM of vincristine for 72 h the proportions change to MDR^High^ = 40% and MDR^Low^ = 60%. For further details please refer to Pisco et al [17].

The image for Fig. 4, “Three-phenotypic breast cancer cell population dynamics with both growth and transition from model simulation,” is incorrect. Please see the corrected here Fig. 4.

**Fig 4:**
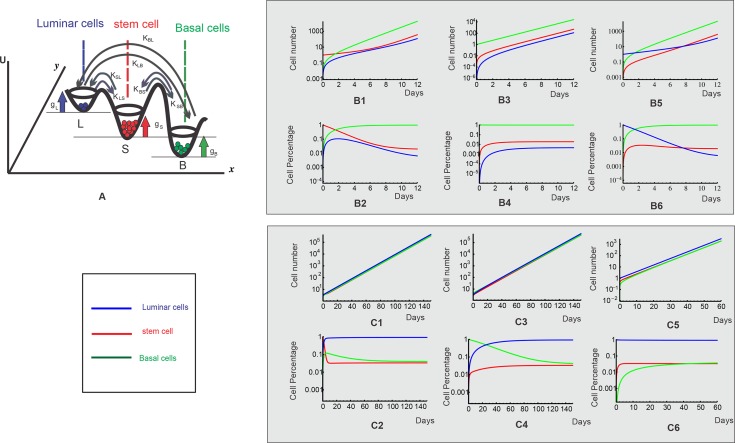
Three-phenotypic breast cancer cell population dynamics with both growth and transition from model simulation. **A.** Illustration of cell growth and transition for breast cancer cell line with three distinct cell phenotypes: luminal cell, basal cell and mammary stem cell. **B1-B6**. After FACS sorting, each isolated subpopulation of cell line SUM159, stem-like, basal and luminal cells, re-equilibrate to the stable cell-state ratio r*. Upper panels are the dynamics for cell numbers of three subpopulations; Lower panels are the dynamics for the cell ratios of three subpopulations. **C1-C6**. After FACS sorting, each isolated subpopulation of cell line SUM149, stem-like, basal and luminal cells, re-equilibrate to the stable cell-state ratio r* Upper panels are the dynamics for cell numbers of three subpopulations; Lower panels are the dynamics for the cell ratios of three subpopulations.

There are errors in the Author Contributions. The correct contributions are: Conceived and designed the experiments: JZ, HQ and SH. Performed the experiments: JZ and AOP. Analyzed the data: JZ and AOP. Contributed reagents/materials/analysis tools: HQ and AOP. Wrote the paper: JZ and AOP. All authors participated in editing the manuscript.
